# Improved prediction of drug-induced liver injury literature using natural language processing and machine learning methods

**DOI:** 10.3389/fgene.2023.1161047

**Published:** 2023-07-17

**Authors:** Jung Hun Oh, Allen Tannenbaum, Joseph O. Deasy

**Affiliations:** ^1^ Department of Medical Physics, Memorial Sloan Kettering Cancer Center, New York, NY, United States; ^2^ Department of Computer Science, Stony Brook University, Stony Brook, NY, United States; ^3^ Department of Applied Mathematics and Statistics, Stony Brook University, Stony Brook, NY, United States

**Keywords:** natural language processing, TF-IDF, Word2vec, drug-induced liver injury, artificial intelligence, machine learning

## Abstract

Drug-induced liver injury (DILI) is an adverse hepatic drug reaction that can potentially lead to life-threatening liver failure. Previously published work in the scientific literature on DILI has provided valuable insights for the understanding of hepatotoxicity as well as drug development. However, the manual search of scientific literature in PubMed is laborious and time-consuming. Natural language processing (NLP) techniques along with artificial intelligence/machine learning approaches may allow for automatic processing in identifying DILI-related literature, but useful methods are yet to be demonstrated. To address this issue, we have developed an integrated NLP/machine learning classification model to identify DILI-related literature using only paper titles and abstracts. For prediction modeling, we used 14,203 publications provided by the Critical Assessment of Massive Data Analysis (CAMDA) challenge, employing word vectorization techniques in NLP in conjunction with machine learning methods. Classification modeling was performed using 2/3 of the data for training and the remainder for test in internal validation. The best performance was achieved using a linear support vector machine (SVM) model on the combined vectors derived from *term frequency-inverse document frequency* (*TF-IDF*) and *Word2Vec*, resulting in an accuracy of 95.0% and an F1-score of 95.0%. The final SVM model constructed from all 14,203 publications was tested on independent datasets, resulting in accuracies of 92.5%, 96.3%, and 98.3%, and F1-scores of 93.5%, 86.1%, and 75.6% for three test sets (T1-T3). Furthermore, the SVM model was tested on four external validation sets (V1-V4), resulting in accuracies of 92.0%, 96.2%, 98.3%, and 93.1%, and F1-scores of 92.4%, 82.9%, 75.0%, and 93.3%.

## 1 Introduction

Drug-induced liver injury (DILI) is a liver disease caused by an adverse drug reaction that can potentially lead to fatal liver failure (David and Hamilton, 2010). Previously published work in the scientific literature on DILI has provided valuable insights for the understanding of hepatotoxicity on causative agents and clinical features as well as drug development (Khoury et al., 2015; Weaver et al., 2020). However, the manual search for relevant scientific literature in PubMed has been proven to be laborious and time-consuming, potentially resulting in low recall (the faction of correctly retrieved documents among all relevant documents) (Richardet et al., 2015). Natural language processing (NLP) techniques have been developed to decipher and understand the meaning of human language by extracting useful information from unstructured text data (Lopez-Ubeda et al., 2022; Undru et al., 2022). In particular, NLP in conjunction with artificial intelligence (AI)/machine learning techniques can be efficiently used to convert words to a representation of real numbers and therefore allow for model building, in an integrated data analysis pipeline (Zhan et al., 2022a; Rathee et al., 2022).

In NLP, two word vectorization techniques are widely employed for feature extraction by mapping words to a corresponding vector of real numbers: word embedding and Bag-of-Words (BoW) with term frequency-inverse document frequency (TF-IDF) weighting (Zhan et al., 2022a). In TF-IDF, each value in the vector indicates the count of words appearing in a document or sentence. Word embedding is a technique to represent words into a dense, low-dimensional space, while preserving the inter-word semantics. The three primary shallow neural network-based word embedding methods include Word2Vec (Mikolov et al., 2013), GloVe (Pennington et al., 2014), and fastText (Facebook, 2016). For text classification, the resulting vectors can be utilized as features in conventional machine learning methods.

Recently, Bidirectional Encoder Representations from Transformers (BERT) was introduced, which is a transformer-based language model designed employing a deep learning architecture (Devlin et al., 2019; Ambalavanan and Devarakonda, 2020). BERT was pre-trained using the entire English Wikipedia and book corpus datasets with a total of 3,300 million words (Yao et al., 2019). BERTBASE has 12 stacked encoder layers with 110 million parameters and 768 hidden units, whereas BERTLARGE has 24 stacked encoder layers with 340 million parameters and 1,024 hidden units (Anggrainingsih et al., 2022). A large number of domain-specific BERT-based language models have been developed, leveraging their own semantic information, that include BioBERT (Lee et al., 2020; Zhu et al., 2020), RadBERT (Yan et al., 2022), SciBERT (Beltagy et al., 2019), ClinicalBERT (Huang et al., 2019), and BlueBERT (Peng et al., 2019).

In the present study, we demonstrate that the combined vectors derived from TF-IDF and Word2Vec in conjunction with machine learning methods can improve performance in predicting DILI-related literature via internal and external validation.

## 2 Materials and methods

### 2.1 Data

The Critical Assessment of Massive Data Analysis (CAMDA) 2022 in collaboration with the Intelligent Systems for Molecular Biology (ISMB) hosted the Literature AI for Drug Induced Liver Injury (DILI) challenge (Zhan et al., 2022b). A curated dataset, consisting of 277,016 DILI annotated papers, was downloaded from the CAMDA website. All the papers were labeled as DILI-related (referred to as “positive samples”) or irrelevant to DILI (referred to as “negative samples”) by a panel of DILI experts. For the CAMDA challenge, the labels for 7,177 DILI-related papers and 7,026 DILI-unrelated papers were released while the labels for the remaining papers (*N* = 262,813) were masked for model assessment and were split into three test sets and four validation sets. In this study, 14,203 papers with positive or negative labels were used for model building. For each paper, the title and abstract were provided and concatenated to be used in modeling.

### 2.2 Word vectorization

To extract and quantify text features from the unstructured literature, we employed two word vectorization techniques: word embedding with Word2Vec implemented in the Gensim (v.4.3.1) Python library (Rehurek and Sojka, 2011) and BoW with TF-IDF weighting (Zhang et al., 2010). Before vectorization, text data were preprocessed by lowercasing, removing punctuations, special characters, white spaces, and standard stop-words followed by lemmatization, employing the spaCy (v.2.3.8) Python library (Honnibal and Montani, 2017). The Word2Vec model was trained using the skip-gram method with a window size of 5 words and a 200-dimensional output vector for each word. The resulting output vectors for all the words in a given paper were averaged to create a single 200-dimensional vector to be used in modeling.

### 2.3 Modeling

**FIGURE 1:**
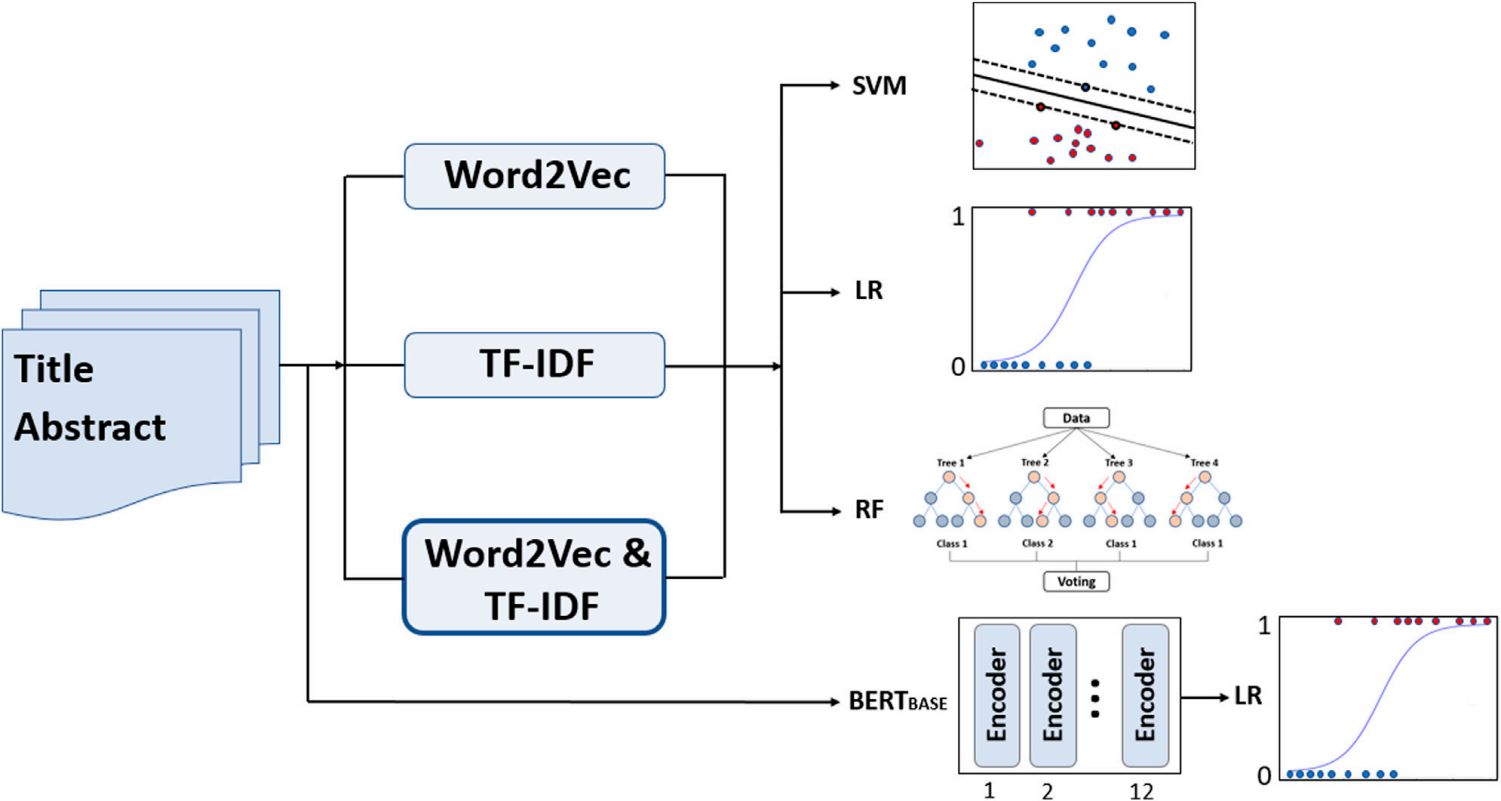
The proposed pipeline of data analysis using natural language processing in conjunction with machine learning methods on 7,177 DILI-related and 7,026 DILI-unrelated papers. The BERT_BASE_ indicates three different BERT_BASE_ models; a general BERT_BASE_ model was pre-trained using the entire English Wikipedia and book corpus datasets with a total of 3,300 million words, and two BlueBERT_BASE_ models were pre-trained on: (1) PubMed abstracts and (2) PubMed abstracts and clinical notes (MIMIC-III). TF-IDF: term frequency-inverse document frequency; SVM: support vector machine; LR: logistic regression; RF: random forest.

Several machine learning methods, including linear support vector machine (SVM), logistic regression, and random forest were then tested using the transformed numerical features obtained from the Word2Vec and TF-IDF models on 7,177 DILI-related and 7,026 DILI-unrelated papers (Figure 1). For SVM modeling, the slack variable (C) was set to 100. For random forest modeling, the number of trees was set to 1,000, the node size was set to 3, and *mtry* was set to the square root of the number of predictors. In addition, the Transformer-based BERT_BASE_ language model and two BlueBERT_BASE_ models pre-trained on: (1) PubMed abstracts and (2) PubMed abstracts and clinical notes (MIMIC-III) were tested for benchmark comparison (Devlin et al., 2018). All BERT models were fine-tuned for five epochs with a learning rate of 1e-5, employing the Transformers (v.4.21.2) Python library.

**FIGURE 2:**
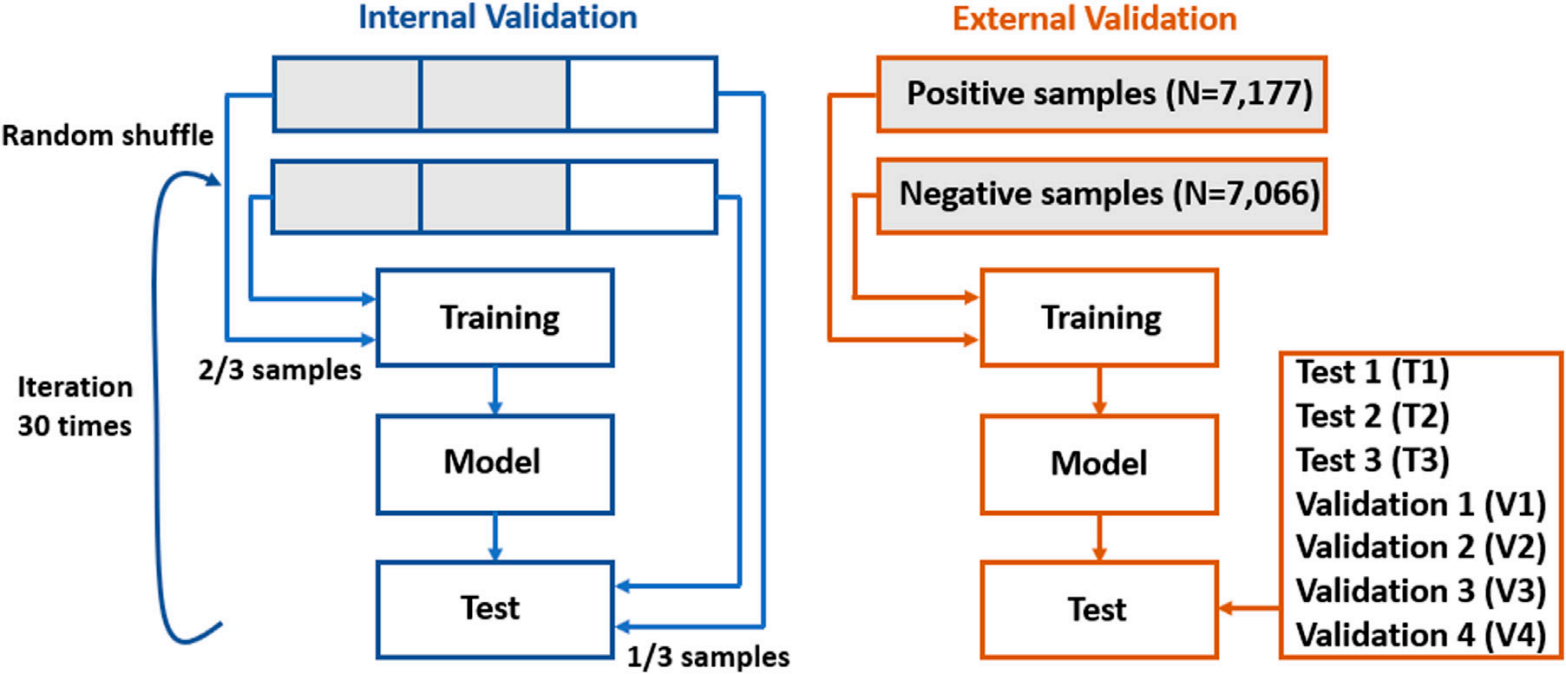
Internal and external validation strategy.

For internal validation in the machine learning modeling, the data of 14,203 samples were randomly stratified into the training (66.7%) and test (33.3%) sets. For the BERT_BASE_ modeling, the data were stratified into the training (56.7%), validation (10%), and test (33.3%) sets. This modeling process was iterated 30 times, and the average accuracy, precision, recall, and F1-score assessed on the test set were reported. For external validation, a final model was built using all the 14,203 samples and was tested on seven independent datasets (in total, *N* = 262,813): three test (T1-T3) and four validation sets (V1-V4). V1, V2, and V3 datasets have an increasing level of imbalance in terms of the number of DILI-related and DILI-unrelated papers, and match with T1, T2, and T3 datasets. In addition, the V4 dataset was used to assess the generalizability of prediction models. Note that the test sets differ from those used in the internal validation. All test and validation sets used in the external validation are independent external data; the difference between them is that there was no restriction in the number of submissions of predicted outcomes in the CAMDA system when we assessed the trained model on the test sets. Figure 2 illustrates the strategy for internal and external validation. All experiments were conducted in a Google Colab Pro+ with codes implemented in Python.

### 2.4 Model explainability

The LIME (v.0.2.0.1) Python library was used for explainable analysis of predictions made by machine learning models, identifying key words and their importance scores (Ribeiro et al., 2016). In addition, cosine similarity in Word2Vec was used to assess the closeness between words.

## 3 Results

### 3.1 Data visualization

**FIGURE 3:**
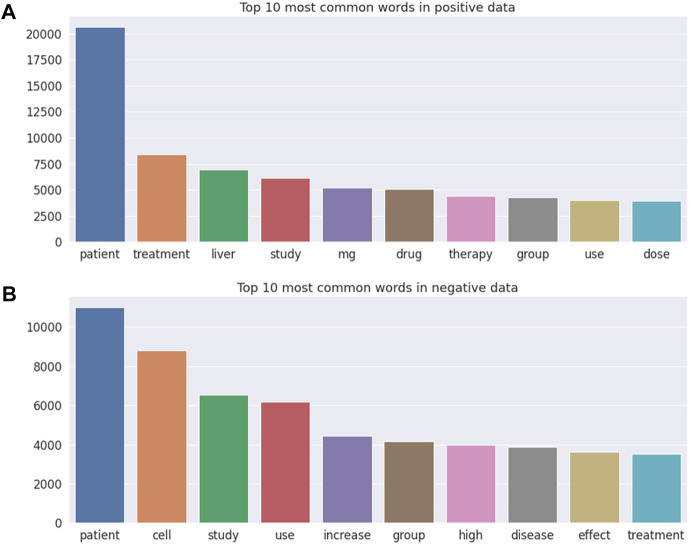
The top 10 most common words in **(A)** DILI-related and **(B)** unrelated literature.

Figure 3 illustrates the top 10 most common words in DILI-related (Figure 3A) and unrelated (Figure 3B) publications, with “patient” being the most frequent word in both DILI-related and unrelated publications. Note that “liver” and drug relevant words including “mg,” “drug,” and “dose” were the frequently occurring words in DILI-related literature, but were not included in the top 10 most common words in DILI-unrelated literature.

**FIGURE 4:**
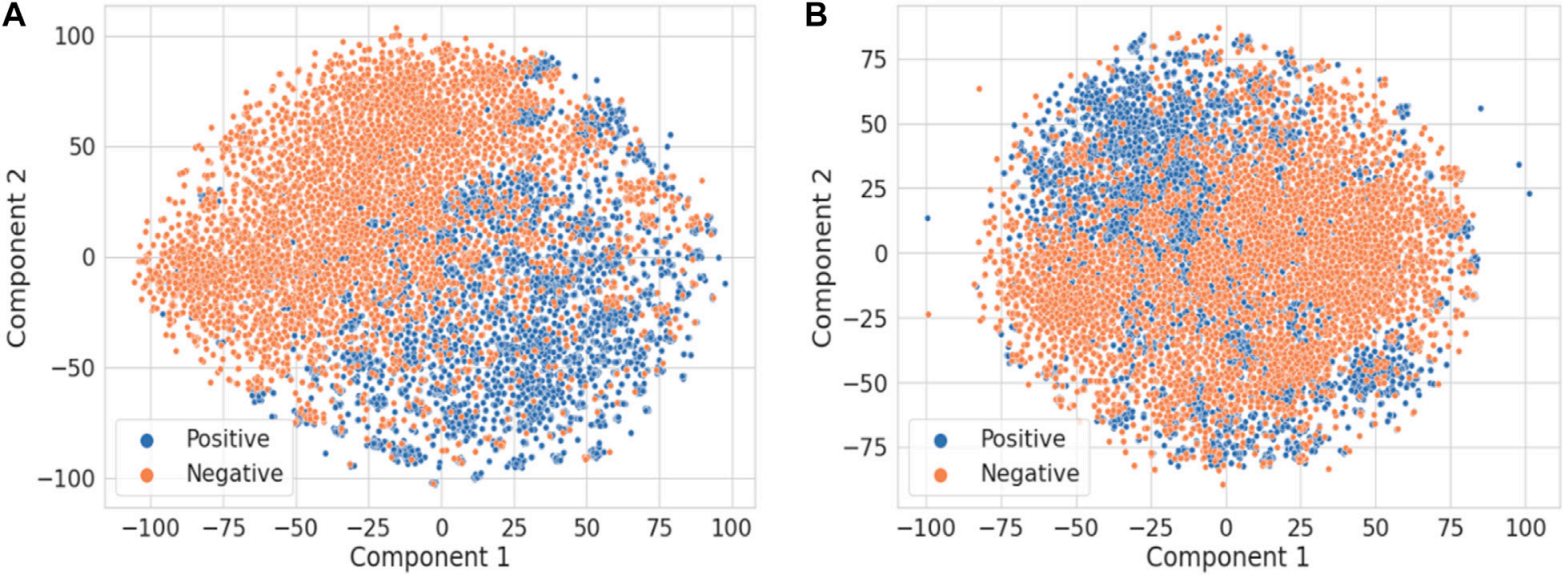
The t-SNE visualization of the TF-IDF vectors obtained using **(A)** the title and abstract and **(B)** only the title of each publication.

Figure 4 illustrates the t-distributed stochastic neighbor embedding (t-SNE) visualization of the TF-IDF vectors obtained using the combined title and abstract (Figure 4A) vs. only the title of each publication (Figure 4B) (van der Maaten and Hinton, 2008). It is not surprising that the abstract has much more information than the title to distinguish DILI-related publications from the ones unrelated to DILI. For comparison, principal component analysis results are shown in Supplementary Figure S1.

### 3.2 Internal validation

**FIGURE 5:**
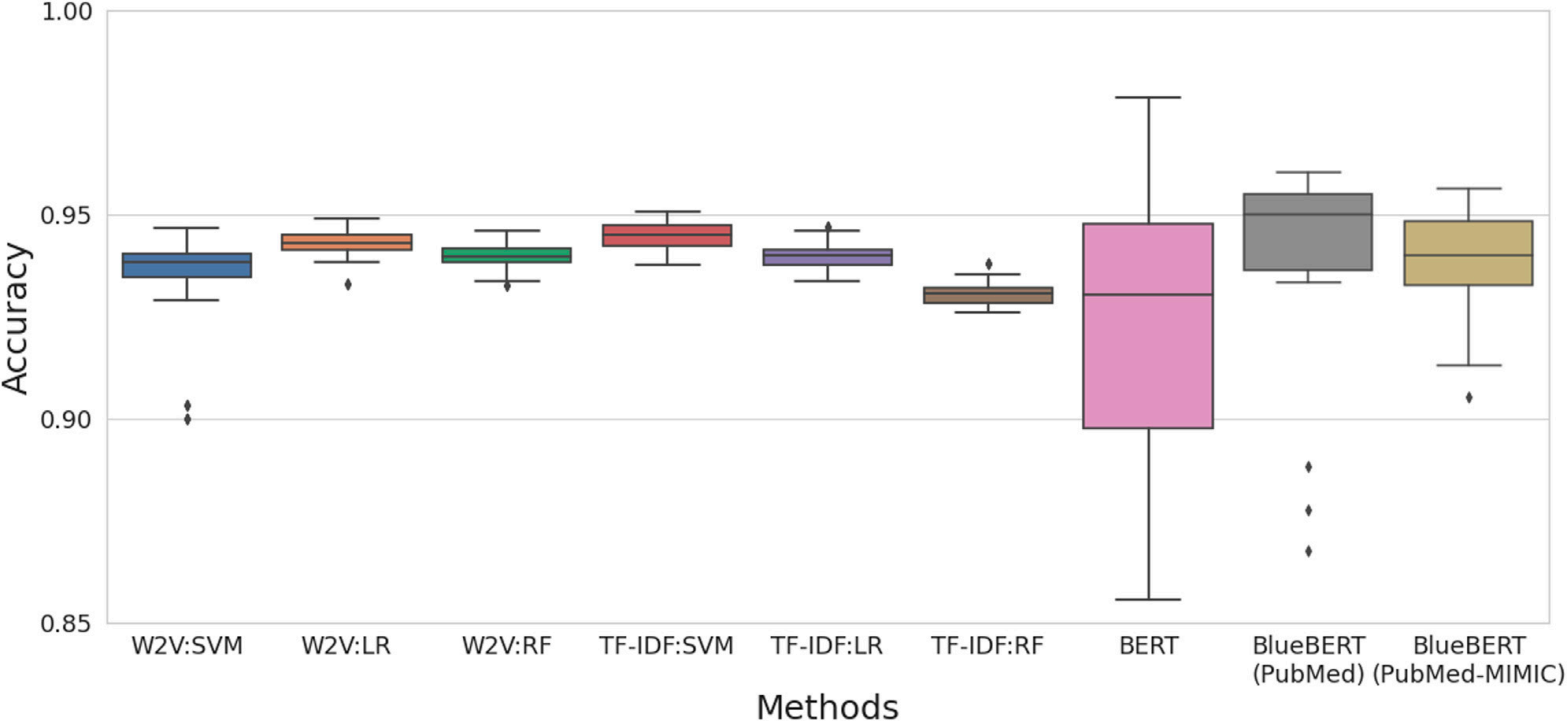
The accuracy of machine learning methods (SVM: support vector machine, LR: logistic regression, and RF: random forest) on test data using vectors derived from two vectorization techniques (W2V: Word2Vec and TF-IDF) in comparison with the BERT models. All tests were conducted on the combined title and abstract.

Figure 5 shows the accuracy of machine learning models on test data after 30 iterations, employing vectors derived from the Word2Vec and TF-IDF models on the combined title and abstract. A linear SVM model using vectors derived from the TF-IDF model achieved the best accuracy of 94.5%. Interestingly, a linear SVM using TF-IDF vectors derived from only the title of publications obtained an accuracy of 88.8%, whereas a linear SVM using TF-IDF vectors derived from only the abstract of publications obtained an accuracy of 94.3%, implying that the information available in the title of publications did not improve performance in classifying DILI-related literature. For the benchmark test, three BERT_BASE_ models were assessed: general BERT, BERT pre-trained on PubMed, and BERT pre-trained on PubMed and MIMIC III. A general BERT model had worse performance in all metrics as compared to other machine learning models, whereas BlueBERT (PubMed) and BlueBERT (PubMed+MIMIC) models achieved much improved predictive performance in comparison with the general BERT model.

In addition, machine learning models were tested on the combined data of Word2Vec and TF-IDF vectors. A linear SVM model achieved the best performance with an accuracy of 95.0%, a precision of 95.3%, a recall of 94.7%, and an F1-score of 95.0% (Table 1). Overall, this approach improved classification performance in all machine learning models compared to the models built on Word2Vec or TF-IDF vectors alone.

**TABLE 1:** The accuracy, precision, recall, and F1-score of machine learning methods on test data using vectors derived from two vectorization techniques. The values in parentheses are the standard deviation. SVM, support vector machine; LR, logistic regression; RF, random forest.

Vectorization	Methods	Accuracy (%)	Precision (%)	Recall (%)	F1-score (%)
Word2Vec	SVM	93.6 (1.0)	94.7 (1.9)	92.5 (3.4)	93.6 (1.2)
	LR	94.3 (0.3)	94.6 (0.5)	94.1 (0.5)	94.4 (0.3)
	RF	94.0 (0.3)	93.9 (0.5)	94.2 (0.5)	94.1 (0.3)
TF-IDF	SVM	94.5 (0.4)	94.8 (0.4)	94.3 (0.5)	94.5 (0.4)
	LR	94.0 (0.3)	94.9 (0.5)	93.1 (0.5)	94.0 (0.3)
	RF	93.1 (0.3)	94.1 (0.5)	92.0 (0.5)	93.0 (0.3)
Word2Vec & TF-IDF	SVM	**95.0 (0.3)**	95.3 (0.4)	94.7 (0.4)	95.0 (0.3)
	LR	94.9 (0.3)	95.1 (0.5)	94.8 (0.4)	94.9 (0.3)
	RF	94.1 (0.3)	93.8 (0.5)	94.6 (0.5)	94.2 (0.3)
BERT models	BERT	91.7 (5.3)	93.9 (5.6)	92.5 (3.0)	92.3 (3.2)
	BERT (PubMed)	93.8 (3.0)	92.3 (5.6)	96.3 (2.8)	94.1 (2.5)
	BERT (PubMed + MIMIC)	93.9 (1.2)	93.9 (2.9)	94.3 (4.2)	94.0 (1.4)

Bold indicates the best accuracy.

### 3.3 External validation

A final linear SVM model was tested on seven independent datasets, resulting in accuracies of 92.5%, 96.3%, and 98.3%, and F1-scores of 93.5%, 86.1%, and 75.6% for three test sets (T1-T3) as shown in Table 2. The SVM model was further tested on four validation sets (V1-V4), resulting in accuracies of 92.0%, 96.2%, 98.3%, and 93.1%, and F1-scores of 92.4%, 82.9%, 75.0%, and 93.3%. Predictive performance on T1-T3 datasets was similar to that on V1-V3 datasets. Note that F1-scores from T1 to T3 (similarly, from V1 to V3) decreased likely due to an increasing level of imbalance in the number of DILI-related and DILI-unrelated papers.

**TABLE 2:** The accuracy, precision, recall, and F1-score of a final model assessed on three test and four validation sets.

Dataset	*N*	Accuracy (%)	Precision (%)	Recall (%)	F1-score (%)
Test 1 (T1)	4,763	92.5	93.5	93.5	93.5
Test 2 (T2)	21,724	96.3	83.3	89.1	86.1
Test 3 (T3)	82,753	98.3	73.4	78.0	75.6
Validation 1 (V1)	6,494	92.0	91.9	92.9	92.4
Validation 2 (V2)	32,814	96.2	77.8	88.8	82.9
Validation 3 (V3)	100,265	98.3	72.7	77.5	75.0
Validation 4 (V4)	14,000	93.1	90.3	96.5	93.3

### 3.4 Model explainability

**FIGURE 6:**
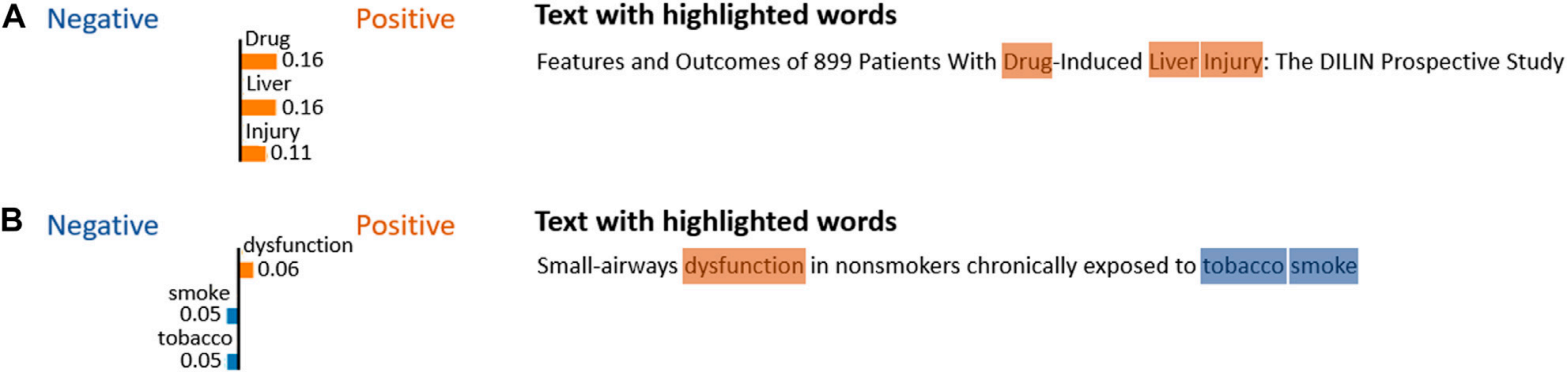
Representative examples for explainable analysis using the LIME Python library. **(A)** DILI-related paper (PMID: 25754159) and **(B)** DILI-unrelated paper (PMID: 7354778).

To explain the predictions made by machine learning models, the LIME Python library was utilized. Figure 6 illustrates two representative examples with a DILI-related paper and a DILI-unrelated paper. The top three words and their importance scores used for prediction are shown in each paper’s title. This analysis is very useful for interpretability of the models, which enables the identification of important words that contribute to the prediction.

**FIGURE 7:**
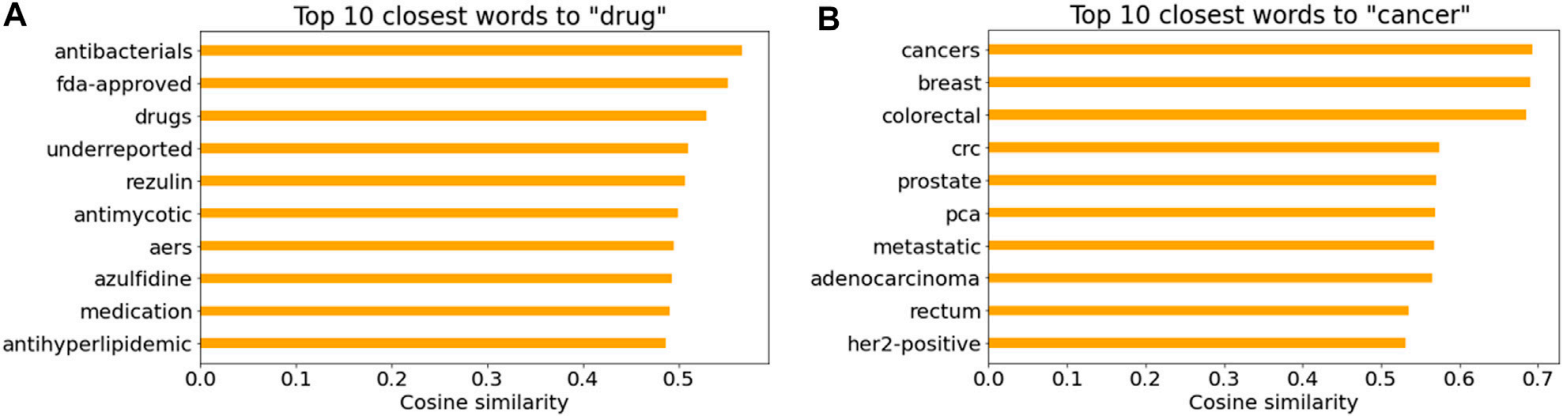
Cosine similarity for **(A)** “drug” and **(B)** “cancer” in the Word2Vec model.

Figures 7A, B illustrate the top 10 closest words to “drug” and “cancer” in the Word2Vec model based on the cosine similarity, respectively. It is not surprising that drug names and cancer-related words were listed. Note that “AERS” in Figure 7A indicates the FDA Adverse Event Reporting System that contains adverse event reports.

## 4 Discussion

To classify DILI-related literature, in this study, we proposed to use the combined vectors derived from Word2Vec and TF-IDF models that are trained using paper titles and abstracts. A linear SVM model using the combined vectors achieved better predictive performance compared to the models using vectors derived from either Word2Vec or TF-IDF alone as well as the BERT-based models. Interestingly, a linear SVM model exploiting only the title of publications obtained an accuracy of 88.8%, suggesting that the title of publications includes concise yet comprehensive information about the content of publications.

For the benchmark test, three BERT_BASE_ models were assessed: general BERT, BlueBERT (PubMed), and BlueBERT (PubMed and MIMIC III). The two BlueBERT models achieved similar predictive performance with accuracies of 93.8% and 93.9% and F1-scores of 94.1% and 94.0%. Not surprisingly, the predictive performance was much better than that of the general BERT with an accuracy of 91.7% and an F1-score of 92.3%. Overall, the BlueBERT models had smaller standard deviation in performance metrices compared to the general BERT, implying the stability of the BlueBERT models. This indicates that it is important to develop domain-specific BERT models for NLP analysis.

## 5 Conclusion

Machine learning methods using vectors derived from NLP text vectorization techniques were developed to classify literature related or unrelated to DILI. Machine learning models trained utilizing the combined data of Word2Vec and TF-IDF vectors improved classification performance as compared to the models trained using either Word2Vec or TF-IDF vectors alone. The developed analysis pipeline allows for easy adaptation to other NLP problems, facilitating the analysis of free-text documents by employing the integration of NLP and machine learning techniques.

## Data Availability

Publicly available datasets were analyzed in this study. This data can be found here: http://camda.info/.
