# Supplementary material for: Improved prediction of drug-induced liver injury literature using natural language processing and machine learning methods
Source: Front Genet. 2023 Jul 17;14:1161047. doi: 10.3389/fgene.2023.1161047 (PMC10390074; doi:10.3389/fgene.2023.1161047)

**Supplementary Figure S1.** Principal component analysis on the TF-IDF vectors obtained using (A) the title and abstract and (B) only the title of each publication.


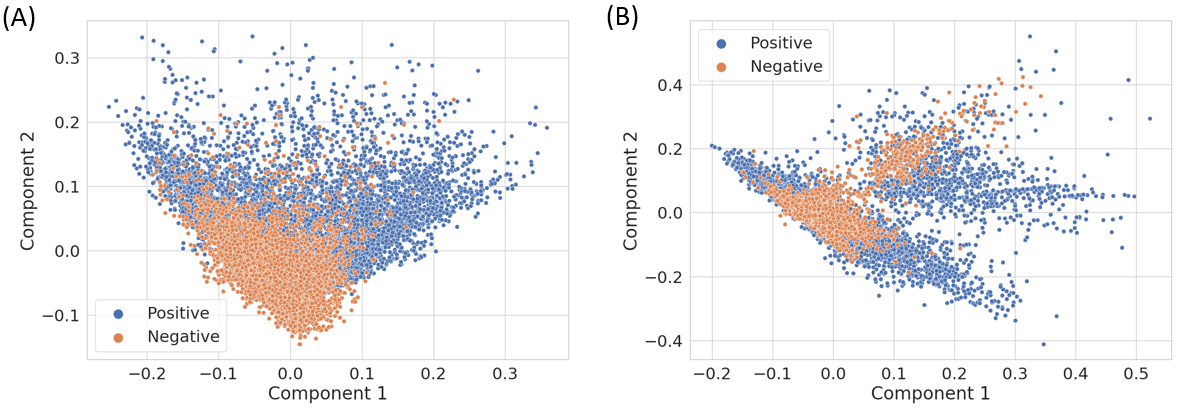

Supplement: Supplementary file 1 [file Table1.DOCX]
